# Book Reviews, Pamphlets, Exchanges

**Published:** 1901-07

**Authors:** 


### *BOOK REVIEWS, PAMPHLETS, EXCHANGES.*

---

Principles of Surgery.—By N. Senn, M.D., Ph.D., LL.D., Professor of Surgery in Rush Medical College in Affiliation with the University of Chicago; Professorial Lecturer on Military Surgery in the University of Chicago; Attending Surgeon to the Presbyterian Hospital; Surgeon-in-Chief to St. Joseph's Hospital; Surgeon-General of Illinois; Late Lieutenant-Colonel of United States Volunteers and Chief of the Operating Staff with the Army in the field during the Spanish-American War. Third Edition. Thoroughly Revised, with 230 Wood Engravings, Half-Tones, and Colored Illustrations. Royal Octavo; pages, xiv.—700. Extra Cloth, $4.50, net; Sheep or Half-Russia, $5.50, net. Delivered. Philadelphia: F. A. Davis Company, Publishers, 1914–16 Cherry Street.

The third is an enlarged and perfected edition of the first. Numerous editions have been made and new chapters added on degeneration and blastomycetic dermatitis. The volume is fully illustrated. The work is already well known and esteemed and there is certainly none more meritorious in the field of surgery.

---

A System of Physiologic Therapeutics. A Practical Exposition of the Methods, Other than Drug-Giving, Useful in the Treatment of the Sick. Edited by Solomon Solis-Cohen, A.M., M.D., Professor of Medicine and Therapeutics in the Philadelphia Polyclinic; Lecturer on Clinical Medicine at Jefferson Medical College, etc. Volume 11, Electrotherapy, by George W. Jacoby, M.D., Consulting Neurologist to the German Hospital, New York City; to the Infirmary for Women and Children, etc. In Two books: Book II, Diagnosis; Therapeutics. Illustrated. Published by P. Blakiston's Son & Co., 1012 Walnut St., Philadelphia, Pa. Price, eleven volumes, $22.00 net.

The object of this system is to treat of the measures other than drugs used in the treatment of disease. The two volumes already published deal very fully with electrophysics and electrotherapeutics. The system gives promise of being a work of great value. The binding and press work on the volumes are exceptionally fine.

